# Distributed Consensus Algorithms in Sensor Networks with Higher-Order Topology

**DOI:** 10.3390/e25081200

**Published:** 2023-08-11

**Authors:** Qianyi Chen, Wenyuan Shi, Dongyan Sui, Siyang Leng

**Affiliations:** 1 School of Information Science and Technology, Fudan University, Shanghai 200433, China; 2 School of Mathematical Sciences, Fudan University, Shanghai 200433, China; 3 Institute of AI and Robotics, Academy for Engineering and Technology, Fudan University, Shanghai 200433, China; syleng@fudan.edu.cn; 4 Research Institute of Intelligent Complex Systems, Fudan University, Shanghai 200433, China

**Keywords:** distributed consensus algorithm, sensor network, higher-order topology, non-Bayesian social learning

## Abstract

Information aggregation in distributed sensor networks has received significant attention from researchers in various disciplines. Distributed consensus algorithms are broadly developed to accelerate the convergence to consensus under different communication and/or energy limitations. Non-Bayesian social learning strategies are representative algorithms for distributed agents to learn progressively an underlying state of nature by information communications and evolutions. This work designs a new non-Bayesian social learning strategy named the hypergraph social learning by introducing the higher-order topology as the underlying communication network structure, with its convergence as well as the convergence rate theoretically analyzed. Extensive numerical examples are provided to demonstrate the effectiveness of the framework and reveal its superior performance when applying to sensor networks in tasks such as cooperative positioning. The designed framework can assist sensor network designers to develop more efficient communication topology, which can better resist environmental obstructions, and also has theoretical and applied values in broad areas such as distributed parameter estimation, dispersed information aggregation and social networks.

## 1. Introduction

Sensor networks are widely deployed in environment for data gathering and monitoring purposes [1,2]. Originally motivated in military surveillance and then popularized to mobile and wireless communication, a large number of low-cost radars, endowed with communication capability, are distributedly located to fulfill tasks such as cooperative positioning and target recognition [3,4,5,6,7]. In typical applications, sensors collaborate to reach a consensus that accurately represents the correct classification of an event or the underlying true state of nature through a predefined communication network that is usually described by a connected graph and suffers from communication constraints [8]. Information processing in sensor networks has thus received significant attention from various disciplines such as computer science, signal processing and control theory [9,10].

The fusion-centric approaches assume each sensor has a communication link to a data fusion center, which is relatively energy consuming in large-scale networks [11]. Distributed consensus algorithms are then broadly developed to accelerate the convergence to consensus under different communication and/or energy limitations [12,13]. Social learning strategies, originating from social networks, are representative algorithms for distributed agents to learn progressively an underlying state of nature by information communications and evolutions [14]. Seminally, Banerjee [15] and Bikhchandani et al. [16] formulate the social learning paradigm in a fully Bayesian manner, which is further comprehensively analyzed and developed by Smith and Sørensen [17]. However, the Bayesian learning framework has a stringent requirement for a priori information and a high computational burden, preventing its usage even in simple networks [18]. Non-Bayesian algorithms are then introduced into social learning [19,20,21,22]; they have been flourishingly developing since the pioneering work of Jadbabaie et al. [14,23,24]. The non-Bayesian social learning framework consists of a *belief aggregation* step and a *Bayesian update* step, dealing with external communication and internal belief updating, respectively. In [25], the original model is extended using logarithmic aggregation, with its convergence and asymptotic normality being proved. Kar et al. [26] design a set of distributed parameter estimation algorithms by combining a consensus step and an innovation step in the update rule and apply them in sensor networks. The non-Bayesian social learning algorithms are subsequently generalized to various network structures. For example, Nedić et al. [27,28] analyze the learning rule for time-varying graphs, and the convergence result of a non-Bayesian learning algorithm for fixed graphs is provided in [29]. Authors in [30] consider the learning rules on weakly connected graphs, and social learning with time-varying weights is studied in [31]. Recently, a novel adaptive social learning strategy has been proposed by Bordignon et al. [32] to address the poor performance under nonstationary conditions. Sui et al. design a parametric social learning framework, introducing an agent stubbornness parameter to trade off the significance between its internal belief and external communication [33]. Notice that consensus networks, also known as opinion dynamics, have been extensively studied for many years [34,35]. Only paying attention to interpersonal communication, opinion dynamics focus on the formation, evolution and convergence of beliefs within a group [36,37]. Plentiful methods are proposed to judge/guarantee convergence in such networks. For example, a negative spectral gap is necessary to guarantee the convergence in the Influence Network (I-net) [38]. However, social learning frameworks proceed further than consensus networks by considering external communication and internal belief updating simultaneously, since they are more aligned with the reality of social networks. The difference also lies in the fact that social learning strategy converges to one of the preconceived hypotheses, while opinion dynamics converge to a certain consensus value.

Undoubtedly, the underlying network structure plays a key role in determining the convergence as well as its rate of the non-Bayesian social learning algorithms [39]. Practically, most sensors are physically restricted by their limited communication bandwidth, in which case they can only communicate with their local neighbors [8]. Moreover, reliability and connectivity are also constraints that the distributed consensus algorithms should address. In certain scenarios, a part of sensors is obstructed or misguided due to environmental factors. These drawbacks call for advanced design of the learning algorithms addressing more realistic circumstances. Real-world complex networks, including ecological networks [40], co-authorship networks [41] and communication networks [42], are proved to contain many-body interactions instead of merely pairwise interactions. In fact, a sum of two-body interactions is not able to capture the difference between the communication of three agents and three separate pairwise communications [43], where the former requires a smaller number of mutual interactions but with similar communication efficiency. For a real-world example in biochemistry, three proteins always interact with each other simultaneously and form a heterotrimer, which functions as a whole [44], and this can not be represented by traditional graphs. Hypergraph structure is then introduced into the non-Bayesian social learning strategies in this work, which extends the existing algorithms to more general situations and exhibits advantages not only in sensor networks, but also in other applications such as social networks.

In this work, we propose new non-Bayesian social learning algorithms based on higher-order topology called hypergraph social learning (HSL), whose convergence as well as the convergence rate are theoretically analyzed. Extensive numerical examples are provided to validate the effectiveness of the algorithms and unveil insights into the relationship between the consensus and hypergraph connectivity/structure. A practical technique for accelerating the convergence is designed and verified. We apply the proposed algorithms to a collective target location problem in sensor networks and analyze the difference with underlying graph and hypergraph structures. The remaining part of this paper is organized as follows: Section 2 provides a full description of the problem settings, reviewing necessary algorithms and proposing our HSL strategies. Section 3 presents sufficient assumptions/lemma and proves the convergence of the proposed algorithms. Section 4 provides extensive numerical examples illustrating the theoretical results and applies the algorithms in sensor networks. The findings are discussed and concluded in Section 5 with possible future work directions.

## 2. Preliminaries and Models

### 2.1. Problem Formulation

We consider a scenario where a group of *n* sensors or agents collaborate in order to arrive at a consensus that accurately represents the correct classification of an event or the underlying true state of nature, denoted as $\theta^{*}$, from a finite set of hypotheses $\Theta =\{\theta_{1},\theta_{2},\cdots ,\theta_{p}\}$. Each agent *i* receives an observation $s_{i,t}$ of an environmental random process at each discrete time step t=1,2,⋯, where $s_{t}={\left(s_{1,t},s_{2,t},\cdots ,s_{n,t}\right)}^{⊤}$ is generated according to a given likelihood function $l(\cdot |\theta^{*})$. The corresponding random variable of agent *i*’s observation at time *t* is denoted as $S_{i,t}$ and $S_{t}={\left(S_{1,t},\cdots ,S_{n,t}\right)}^{⊤}$. Here, each $S_{i,t}$ has its individual observation space $S_{i}$ and is independently and identically distributed with respect to *t*.

The signal structure of agent *i* for state θ is described by probability distribution $l_{i}\left(\cdot |\theta \right)$. In these settings, $l_{i}\left(s_{i,t}|\theta \right)$ characterizes the probability that signal $s_{i,t}$ can be observed by agent *i* at time *t* when it believes θ is the true state. It is required that $l_{i}\left(\cdot |\theta^{*}\right)$ coincides with the *i*th marginal distribution of $l(\cdot |\theta^{*})$, which thus also describes the probability distribution of random variable $S_{i,t}$.

Usually, the agents interact with each other in a networked fashion, which is conventionally modelled by a directed graph describing pairwise interactions [45]. Due to a limited communication bandwidth, reliability and connectivity in a realistic scenario, most sensors are of the entailed constraint type to communicate with their local neighbors, and only a small number of delegates are endowed with mediate communication capacity. This brings in the need for higher-order topological structures that can capture the essence of local multi-agent interactions and centralized mediate communications [46,47]. In this work, hypergraph structure is introduced into the non-Bayesian social learning framework. The designed algorithms are demonstrated to significantly reduce the number of mutual interactions among sensors. A hypergraph is represented as H=(V,E). The set of vertices V={1,2,⋯,n} denotes the *n* agents, and $E=\{e_{j}|j=1,2,\cdots ,m\}$ is the set of *m* hyperedges. Unlike a graph, a hyperedge may connect more than two nodes, leading to a different aggregation mechanism. We denote $A={\left(a_{ji}\right)}_{m\times n}$ as the weight matrix with its row vector representing the weights of nodes in a single hyperedge. Moreover, $B={\left(b_{ij}\right)}_{n\times m}$ denotes the weight matrix characterizing the weights of each node with respect to every hyperedge that it belongs to. Both *A* and *B* are assumed to be row stochastic, i.e., $\sum_{i=1}^{n}a_{ji}=1,\forall j=1,\cdots ,m$, and $a_{ji}>0$ if $i\in e_{j}$, $\sum_{j=1}^{m}b_{ij}=1,\forall i=1,\cdots ,n$, and $b_{ij}>0$ if $i\in e_{j}$.

The *belief* of agent *i* at time *t* is denoted as $\mu_{i,t}$, which is a probability distribution over the set of possible states Θ, i.e., $\sum_{k=1}^{p}\mu_{i,t}\left(\theta_{k}\right)=1,\forall i=1,\cdots ,n,\forall t=0,1,\cdots$. Here, $\mu_{i,0}$ represents the *initial belief* of agent *i*. We further define the *belief of hyperedge* $e_{j}$ at time *t* as $\nu_{j,t}$ to record the belief aggregation from nodes to hyperedges, which is described detailedly in the following section.

### 2.2. Hypergraph Social Learning

Traditional social learning generally consists of two key procedures at each time step for agents to update their beliefs, i.e., the Bayesian update step and the step of aggregating neighbor beliefs. Taking different orders of these two steps leads to the two basic social learning strategies called the LoAB (Logarithmic Aggregation and then Bayesian update) and the BLoA (Bayesian update and then Logarithmic Aggregation), respectively [48]. Notice that the hypergraph structure broadens the concept of neighbors, inducing the following new belief aggregation approaches.

At time step t+1, the purpose of the Bayesian update step is to update each agent *i*’s prior belief $\mu_{i,t}$ using environmental observation $s_{i,t+1}$ and obtain its posterior belief ${\tilde{\mu }}_{i,t+1}$. This process can also be described as solving an optimization problem as follows:

(1) $${\tilde{\mu }}_{i,t+1}=\underset{f\in P(\Theta )}{\arg\min}\left\{D_{\mathrm{KL}}\left(f\;\|\;\mu_{i,t}\right)-\sum_{\theta \in \Theta }f\left(\theta \right)\log\left(l_{i}\left(s_{i,t+1}|\theta \right)\right)\right\},$$

where $D_{\mathrm{KL}}\left(\cdot \|\cdot \right)$ is the Kullback–Leibler divergence (KL divergence) between two probability distributions. For instance, the first term on r.h.s. of (1) is $D_{\mathrm{KL}}\left(f\;\|\;\mu_{i,t}\right)=\sum_{\theta \in \Theta }f\left(\theta \right)\log\frac{f(\theta )}{\mu_{i,t}\left(\theta \right)}$, describing the difference between the prior belief and the posterior belief. The second term on r.h.s. of (1) describes the maximum likelihood estimation given the latest observation $s_{i,t+1}$. By directly solving the optimization problem (1) with the Lagrange multiplier method, we obtain the update formula for the posterior belief as follows:

(2) $${\tilde{\mu }}_{i,t+1}\left(\theta \right)=\frac{\mu_{i,t}\left(\theta \right)l_{i}\left(s_{i,t+1}|\theta \right)}{\sum_{\theta^{\prime }\in \Theta }\mu_{i,t}\left(\theta^{\prime }\right)l_{i}\left(s_{i,t+1}|\theta^{\prime }\right)}.$$

Inspired by the aggregation mechanism which is always used in Hypergraph Neural Networks (HGNN) [49,50], two steps are needed to achieve belief aggregation in hypergraph structures. The first step called *Node-to-Edge* is performed to aggregate the beliefs of all nodes in each hyperedge to form the defined belief of the hyperedge $\nu_{j,t+1}$. Then, the beliefs of hyperedges are aggregated to the nodes that are contained in their intersections, according to the weights in *B*, and this procedure is called *Edge-to-Node*. This two-step aggregation mechanism is consistent with the real-world prototype. For example, in a scenario of group discussions, participants exchange opinions to reach consensus within the group. If a person participates in multiple group meetings, they then update the personal belief by aggregating multiple consensus from different groups.

Practically, only performing a Bayesian update is not always feasible due to the identifiable problem that the agents encounter. This issue arises when the agents cannot differentiate between certain states based on their individual knowledge. Conversely, relying solely on the aggregation procedure poses a hindrance to agents in obtaining environmental information, consequently impeding their ability to reach consensus on the true state. By combining the Bayesian update step and the aggregation steps in hypergraphs in different orders, we derive two new hypergraph social learning algorithms, i.e.,

(a) HSL-NEB (Hypergraph Social Learning: Node-to-Edge, Edge-to-Node and then Bayesian update):

**Step 1** (Node-to-Edge).
  (3) $$\nu_{j,t+1}\left(\theta \right)=\frac{\exp(\sum_{i=1}^{n}a_{ji}\log\mu_{i,t}\left(\theta \right))}{\sum_{\theta^{\prime }\in \Theta }\exp\left(\sum_{i=1}^{n}a_{ji}\log\mu_{i,t}\left(\theta^{\prime }\right)\right)},\forall \theta \in \Theta ;$$
**Step 2** (Edge-to-Node).
  (4) $${\tilde{\mu }}_{i,t+1}\left(\theta \right)=\frac{\exp(\sum_{j=1}^{m}b_{ij}\log\nu_{j,t+1}\left(\theta \right))}{\sum_{\theta^{\prime }\in \Theta }\exp\left(\sum_{j=1}^{m}b_{ij}\log\nu_{j,t+1}\left(\theta^{\prime }\right)\right)},\forall \theta \in \Theta ;$$
**Step 3** (Bayesian update).
  (5) $$\mu_{i,t+1}\left(\theta \right)=\frac{{\tilde{\mu }}_{i,t+1}\left(\theta \right)l_{i}\left(s_{i,t+1}|\theta \right)}{\sum_{\theta^{\prime }\in \Theta }{\tilde{\mu }}_{i,t+1}\left(\theta^{\prime }\right)l_{i}\left(s_{i,t+1}|\theta^{\prime }\right)},\forall \theta \in \Theta .$$

(b) HSL-BNE (Hypergraph Social Learning: Bayesian update, Node-to-Edge and then Edge-to-Node):

**Step 1** (Bayesian update).
  (6) $${\tilde{\mu }}_{i,t+1}\left(\theta \right)=\frac{\mu_{i,t}\left(\theta \right)l_{i}\left(s_{i,t+1}|\theta \right)}{\sum_{\theta^{\prime }\in \Theta }\mu_{i,t}\left(\theta^{\prime }\right)l_{i}\left(s_{i,t+1}|\theta^{\prime }\right)},\forall \theta \in \Theta ;$$
**Step 2** (Node-to-Edge).
  (7) $$\nu_{j,t+1}\left(\theta \right)=\frac{\exp(\sum_{i=1}^{n}a_{ji}\log{\tilde{\mu }}_{i,t+1}\left(\theta \right))}{\sum_{\theta^{\prime }\in \Theta }\exp\left(\sum_{i=1}^{n}a_{ji}\log{\tilde{\mu }}_{i,t+1}\left(\theta^{\prime }\right)\right)},\forall \theta \in \Theta ;$$
**Step 3** (Edge-to-Node).
  (8) $$\mu_{i,t+1}\left(\theta \right)=\frac{\exp(\sum_{j=1}^{m}b_{ij}\log\nu_{j,t+1}\left(\theta \right))}{\sum_{\theta^{\prime }\in \Theta }\exp\left(\sum_{j=1}^{m}b_{ij}\log\nu_{j,t+1}\left(\theta^{\prime }\right)\right)},\forall \theta \in \Theta .$$

## 3. Assumptions and Results

As widely discussed in previous works of social learning, we care about the convergence of the algorithms as well as the rate of convergence. The following assumptions are required to ensure the convergence of our HSL strategies:

**Assumption 1** (Communication network)**.** *Denote* $C={\left(c_{ij}\right)}_{n\times n}=BA$*, where* $A={\left(a_{ji}\right)}_{m\times n}$ *and* $B={\left(b_{ij}\right)}_{n\times m}$*. The matrix C satisfies that it is the transition matrix of an irreducible, aperiodic Markov chain of finite states.*

To satisfy the assumption, hypergraph H should be a connected hypergraph without isolated vertices (a hypergraph is connected if for any pair of vertices, there is a path which connects these vertices; see Appendix A for detailed explanations). We recall the following lemma [51]:

**Lemma 1.**  *If a Markov chain of finite states is irreducible, then it has a unique stationary distribution π. Let C be the transition matrix of the Markov chain and further suppose it is aperiodic; then, we have $\lim_{k\to \infty }{\left[C^{k}\right]}_{ij}=\pi_{j}$, for 1⩽i,j⩽n.*

The stationary distribution π can be interpreted as the normalized left eigenvector of *C* with respect to eigenvalue 1, which is known as the *eigenvector centrality* in related literatures. The Perron–Frobenius theorem ensures that all components of π are strictly positive.

**Assumption 2** (Belief and signal structure)**.** *For all agents i=1,2,⋯,n,*

*(a) they have positive initial beliefs on all states, i.e., $\mu_{i,0}\left(\theta \right)>0$ for all θ∈Θ;*

*(b) they have positive signal structures, i.e., $l_{i}\left(s_{i}|\theta \right)>0$ for all $s_{i}\in S_{i}$ and θ∈Θ.*

Notice that if the initial belief of agent *i* on state θ is zero, following our HSL algorithms, its belief remains at zero all the time. In this case, θ is meaningless for agent *i*, and we thus eliminate the situation by imposing Assumption 2a. For the signal structures and Assumption 2b, the same explanation can be applied.

Two states, $\theta_{j}$ and $\theta_{k}$, are called *observationally equivalent* for agent *i* if $l_{i}\left(s_{i}|\theta_{j}\right)=l_{i}\left(s_{i}|\theta_{k}\right),\forall s_{i}\in S_{i}$, in which case agent *i* is not able to distinguish these states only with its own information. Moreover, the true state is called *globally identifiable* if the set $\Theta^{*}=\overset{n}{\underset{i=1}{⋂}}\Theta_{i}^{*}$ has only one element $\theta^{*}$, where $\Theta_{i}^{*}=\left\{\theta \in \Theta |l_{i}\left(s_{i}|\theta \right)=l_{i}\left(s_{i}|\theta^{*}\right),\forall s_{i}\in S_{i}\right\}$. This concept can be intuitively explained. If state $\hat{\theta }$ is observationally equivalent to $\theta^{*}$ for all agents, i.e., $\Theta^{*}=\left\{\theta^{*},\hat{\theta }\right\}$, then the two states are exactly the same from the view of all agents and they cannot identify the true state progressively and collectively, which, in addition, induces.

**Assumption 3** (Globally identifiable)**.** *The true state $\theta^{*}$ is globally identifiable.*

Under this assumption, for all $\theta \neq \theta^{*}$, there exists at least agent *i* satisfying the fact that $D_{\mathrm{KL}}\left(l_{i}\left(\cdot |\theta^{*}\right)\;\|\;l_{i}\left(\cdot |\theta \right)\right)$ is strictly positive.

In the following, we denote $K_{i}\left(\theta^{*},\theta \right)=D_{\mathrm{KL}}\left(l_{i}\left(\cdot |\theta^{*}\right)\;\|\;l_{i}\left(\cdot |\theta \right)\right)$ and define a probability triple $\left(\Omega ,F,P^{*}\right)$, where $\Omega =\{\omega |\omega =\left(s_{1},s_{2},\cdots \right)\}$, F is the σ-algebra generated by the observations, and $P^{*}$ is the probability measure induced by paths in Ω, i.e., $P^{*}=\prod_{t=1}^{\infty }l\left(\cdot |\theta^{*}\right)$. $E^{*}\left[\cdot \right]$ is used to denote the expectation operator associated with probability measure $P^{*}$. Now, we can state the main results describing the convergence of the HSL strategies.

**Theorem 1.**  *Under Assumptions 1–3, the update rules (3)–(5) and (6)–(8) satisfy the following properties:*

(9) $$\lim_{t\to \infty }\frac{1}{t}\log\frac{\mu_{i,t}\left(\theta \right)}{\mu_{i,t}\left(\theta^{*}\right)}=-\sum_{j=1}^{n}\pi_{j}K_{j}\left(\theta^{*},\theta \right),\;\forall \theta \neq \theta^{*}$$

*and*

(10) $$\lim_{t\to \infty }\mu_{i,t}\left(\theta^{*}\right)=1,\;P^{*}-a.s.,\;\forall i=1,\cdots ,n.$$

**Proof.**  We consider the update rule (3)–(5) first. For each agent *i* and $\theta \neq \theta^{*}$, we have

$$\begin{array}{cc} \log\frac{\mu_{i,t+1}\left(\theta \right)}{\mu_{i,t+1}\left(\theta^{*}\right)} & =\log\frac{{\tilde{\mu }}_{i,t+1}\left(\theta \right)}{{\tilde{\mu }}_{i,t+1}\left(\theta^{*}\right)}+\log\frac{l_{i}\left(s_{i,t+1}|\theta \right)}{l_{i}\left(s_{i,t+1}|\theta^{*}\right)}\\ \; & =\sum_{j=1}^{m}b_{ij}\sum_{k=1}^{n}a_{jk}\log\frac{\mu_{k,t}\left(\theta \right)}{\mu_{k,t}\left(\theta^{*}\right)}+\log\frac{l_{i}\left(s_{i,t+1}|\theta \right)}{l_{i}\left(s_{i,t+1}|\theta^{*}\right)}. \end{array}$$

By denoting $\varphi_{i,t+1}\left(\theta \right)=\log\frac{\mu_{i,t+1}\left(\theta \right)}{\mu_{i,t+1}\left(\theta^{*}\right)}$ and $L_{i,t+1}\left(\theta \right)=\log\frac{l_{i}\left(s_{i,t+1}|\theta \right)}{l_{i}\left(s_{i,t+1}|\theta^{*}\right)}$, the above equation simplifies to

(11) $$\varphi_{i,t+1}\left(\theta \right)=\sum_{j=1}^{m}b_{ij}\sum_{k=1}^{n}a_{jk}\varphi_{k,t}\left(\theta \right)+L_{i,t+1}\left(\theta \right).$$

We rewrite (11) in the matrix form:

$$\varphi_{t+1}\left(\theta \right)=BA\varphi_{t}\left(\theta \right)+L_{t+1}\left(\theta \right).$$

Then, it follows that

$$\begin{array}{cc} \frac{1}{t}\varphi_{t+1}\left(\theta \right) & =\frac{1}{t}BA\varphi_{t}\left(\theta \right)+\frac{1}{t}L_{t+1}\left(\theta \right)\\ \; & =\frac{1}{t}BA\left(BA\varphi_{t-1}\left(\theta \right)+L_{t}\left(\theta \right)\right)+\frac{1}{t}L_{t+1}\left(\theta \right)=\cdots \\ \; & =\frac{1}{t}{\left(BA\right)}^{t+1}\varphi_{0}\left(\theta \right)+\frac{1}{t}\sum_{k=1}^{t}{\left(BA\right)}^{k}L_{t+1-k}\left(\theta \right)+\frac{1}{t}L_{t+1}\left(\theta \right). \end{array}$$

Using the notation in Assumption 1, the above equation can be written as

(12) $$\frac{1}{t}\varphi_{t+1}\left(\theta \right)=\frac{1}{t}C^{t+1}\varphi_{0}\left(\theta \right)+\frac{1}{t}\sum_{k=1}^{t}C^{k}L_{t+1-k}\left(\theta \right)+\frac{1}{t}L_{t+1}\left(\theta \right),$$

where *C* is an n×n matrix. The assumptions admit that the first and the third terms on the right hand side of (12) converge to zero as t→∞. And the second term can be deformed as

(13) $$\begin{array}{cc} \frac{1}{t}\sum_{k=1}^{t}C^{k}L_{t+1-k}\left(\theta \right)= & \frac{1}{t}\sum_{k=1}^{t}\left(C^{k}-1_{n}\pi \right)L_{t+1-k}\left(\theta \right)\\ \; & +\frac{1}{t}\sum_{k=1}^{t}1_{n}\pi \left(L_{t+1-k}\left(\theta \right)+K\left(\theta^{*},\theta \right)\right)-\frac{1}{t}\sum_{k=1}^{t}1_{n}\pi K\left(\theta^{*},\theta \right), \end{array}$$

where $1_{n}$ is an *n*-dimensional column vector of ones. From Lemma 1, we know that $\lim_{k\to \infty }C^{k}=1_{n}\pi$. Noticing that all elements of $C^{k}\left(k=1,2,\cdots \right)$ are bounded, the first term on the right hand side of (13) converges to zero as t→∞. Moreover,

$$\begin{array}{cc} E^{*}\left[L_{i,t}\left(\theta \right)\right] & =E^{*}\left[\log\frac{l_{i}\left(s_{i,t}|\theta \right)}{l_{i}\left(s_{i,t}|\theta^{*}\right)}\right]=\int_{s\in S_{i}}l_{i}\left(s|\theta^{*}\right)\log\frac{l_{i}\left(s|\theta \right)}{l_{i}\left(s|\theta^{*}\right)}ds\\ \; & =-D_{\mathrm{KL}}\left(l_{i}\left(\cdot |\theta^{*}\right)\;\|\;l_{i}\left(\cdot |\theta \right)\right)=-K_{i}\left(\theta^{*},\theta \right). \end{array}$$

Using the Kolmogorov’s strong law of large numbers, it follows that

$$\frac{1}{t}\sum_{k=1}^{t}L_{t+1-k}\left(\theta \right)-\frac{1}{t}\sum_{k=1}^{t}E^{*}\left[L_{t+1-k}\left(\theta \right)\right]\to 0,\;P^{*}-a.s.,$$

as t→∞, which leads to

$$\lim_{t\to \infty }\frac{1}{t}\sum_{k=1}^{t}1_{n}\pi \left(L_{t+1-k}\left(\theta \right)+K\left(\theta^{*},\theta \right)\right)=0,\;P^{*}-a.s..$$

Now, (13) provides

(14) $$\lim_{t\to \infty }\frac{1}{t}\sum_{k=1}^{t}C^{k}L_{t+1-k}\left(\theta \right)=-1_{n}\pi K\left(\theta^{*},\theta \right),\;P^{*}-a.s..$$

Therefore, property (9) holds, which can be directly induced from (12) and (14). Moreover, it follows that with probability one, for any ϵ>0, there exists an integer *T* such that ∀t>T and $\forall \theta \neq \theta^{*}$,

$$\left|\frac{1}{t}\log\frac{\mu_{i,t}\left(\theta \right)}{\mu_{i,t}\left(\theta^{*}\right)}+\sum_{j=1}^{n}\pi_{j}K_{j}\left(\theta^{*},\theta \right)\right|<\epsilon .$$

Noticing that $\sum_{\theta \neq \theta^{*}}\mu_{i,t}\left(\theta \right)=1-\mu_{i,t}\left(\theta^{*}\right)$, we have

$$\frac{1}{1+\sum_{\theta \neq \theta^{*}}\exp((\epsilon -\sum_{j=1}^{n}\pi_{j}K_{j}\left(\theta^{*},\theta \right))t)}<\mu_{i,t}\left(\theta^{*}\right)⩽1.$$

Letting t→∞, property (10) is then proved because of the arbitrary selection of ϵ.

For update rules (6)–(8), we can reach the same conclusions (9) and (10) via similar analysis.    □

Theorem 1 indicates that all agents interacting in the hypergraph structure eventually learn the underlying true state as long as the assumptions are satisfied. We provide extensive examples to validate the theoretical analyses and unveil more insights in the next section. Moreover, the following corollary describing the convergence rate can be obtained directly from Theorem 1:

**Corollary 1.**  *Under Assumptions 1–3, the update rules (3)–(5) and (6)–(8) satisfy that for all i=1,2,⋯,n and all $\theta \neq \theta^{*}$,*

$$\lim_{t\to \infty }\mu_{i,t}\left(\theta \right)⩽\exp\left(-\alpha_{\theta }t\right),\;P^{*}-a.s.,$$

*where $\alpha_{\theta }=\sum_{j=1}^{n}\pi_{j}K_{j}\left(\theta^{*},\theta \right)$.*

In the following, the set of hyperedges where node *i* resides is denoted as $d_{i}$. We use $\left|e_{j}\right|$ to denote the number of nodes being connected in the *j*th hyperedge and use $\left|d_{i}\right|$ to denote the number of hyperedges where the *i*-th node resides. According to Assumption 1 and Lemma 1, C=BA has a unique stationary distribution $\pi =\left(\pi_{1},\pi_{2},\cdots ,\pi_{n}\right)$, i.e., πC=π. Under specific settings of the weight matrices *A* and *B*, we have further results summarized in the following remark:

**Remark 1.**  *If we consider a special case of A and B as follows,*

$$a_{ji}=\left\{\begin{array}{cc} \frac{1}{\left|e_{j}\right|}, & i\in e_{j},\\ 0, & \mathrm{otherwise}, \end{array}\right.\;b_{ij}=\left\{\begin{array}{cc} \frac{1}{\left|d_{i}\right|}, & i\in e_{j},\\ 0, & \mathrm{otherwise}, \end{array}\right.$$

*we have $\pi_{i}=\frac{|d_{i}|}{\sum_{l=1}^{n}\left|d_{l}\right|}$.*

**Proof.**  From the definitions, it is obvious that *A* and *B* are both row-stochastic matrices. It follows that C=BA is also row stochastic, i.e.,

$$\sum_{j=1}^{n}c_{ij}=1,\;\forall i=1,\cdots ,n.$$

The above equation can be rewritten as

(15) $$\sum_{j=1}^{n}\sum_{e_{k}\in d_{i}\cap d_{j}}b_{ik}a_{kj}=\sum_{j=1}^{n}\sum_{e_{k}\in d_{i}\cap d_{j}}\frac{1}{\left|d_{i}\right|}\frac{1}{\left|e_{k}\right|}=1,\;\forall i=1,\cdots ,n.$$

Denoting $\pi^{\prime }=\pi C=\pi BA$, it follows that

(16) $$\pi_{k}^{\prime }=\sum_{i=1}^{n}\pi_{i}c_{ik}=\sum_{i=1}^{n}\pi_{i}\sum_{j=1}^{m}b_{ij}a_{jk}=\sum_{i=1}^{n}\pi_{i}\sum_{e_{j}\in d_{i}\cap d_{k}}\frac{1}{\left|d_{i}\right|}\frac{1}{\left|e_{j}\right|}.$$

Substituting $\pi_{i}=\frac{|d_{i}|}{\sum_{l=1}^{n}\left|d_{l}\right|}$ into the above equation provides

(17) $$\pi_{k}^{\prime }=\frac{1}{\sum_{l=1}^{n}\left|d_{l}\right|}\sum_{i=1}^{n}\left|d_{i}\right|\sum_{e_{j}\in d_{i}\cap d_{k}}\frac{1}{\left|d_{i}\right|}\frac{1}{\left|e_{j}\right|}=\frac{1}{\sum_{l=1}^{n}\left|d_{l}\right|}\sum_{i=1}^{n}\sum_{e_{j}\in d_{i}\cap d_{k}}\frac{1}{\left|e_{j}\right|}.$$

From (15) and (17) and the uniqueness of the stationary distribution, we obtain

(18) $$\pi_{k}^{\prime }=\frac{|d_{k}|}{\sum_{l=1}^{n}\left|d_{l}\right|}=\pi_{k}.$$

  □

Remark 1 indicates that increasing the degree $|d_{i}|$ of the *i*th node also raises the value of $\pi_{i}$, demonstrating a greater importance of the role of node *i*. This mechanism provides us with viable solutions to accelerate the convergence rate by adjusting the hypergraph structure on nodes that have high eigenvector centrality, which is shown in the following examples.

## 4. Numerical Examples and Applications

Here, we provide several numerical examples demonstrating the above theoretical analyses and apply the HSL algorithms in sensor networks.

### 4.1. Hypergraph Connectivity vs. Convergence

**Figure 1:**
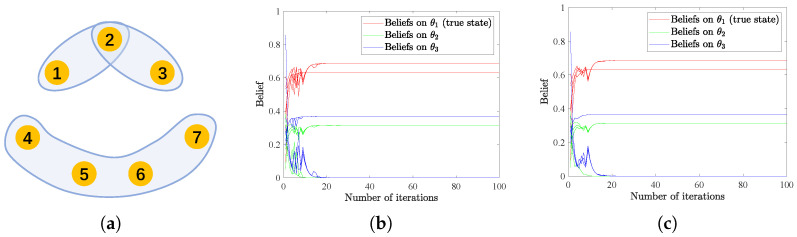

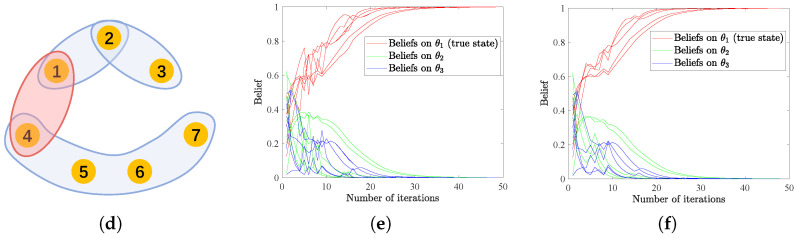
(**a**,**d**) The unconnected and connected hypergraph structures used in this example. (**b**,**c**) The evolution of beliefs on all possible states for agents interacting in structure of (**a**), with (**b**,**c**) denoting the results of HSL-NEB and HSL-BNE, respectively. (**e**,**f**) The evolution of beliefs on all possible states for agents interacting in structure of (**d**), with (**e**,**f**) denoting the results of HSL-NEB and HSL-BNE, respectively.

We first demonstrate the effectiveness of the HSL algorithms in reaching distributed consensus and the relationship with the underlying hypergraph connectivity. The structure of an unconnected hypergraph consisting of seven vertices and three hyperedges is depicted in Figure 1a. The non-zero values in the weight matrices *A* and *B* are randomly generated from (0,1) and then normalized to satisfy the row-stochastic assumption. We assume there are three possible states $\Theta =\{\theta_{1},\theta_{2},\theta_{3}\}$, with $\theta_{1}$ being the true state. The initial beliefs are also uniformly generated from interval (0,1) and subject to $\sum_{k=1}^{3}\mu_{i,0}\left(\theta_{k}\right)=1,\forall i=1,\cdots ,7$. Signals are assumed to be generated at each time from set {H,T} and according to the probability distribution of $l(H|\theta^{*})=0.2$ and $l(T|\theta^{*})=0.8$. Moreover, signal structures are set as $l_{i}\left(H|\theta_{1}\right)=l_{i}\left(H|\theta_{2}\right)=0.2$, $l_{i}\left(T|\theta_{1}\right)=l_{i}\left(T|\theta_{2}\right)=0.8$, $l_{i}\left(H|\theta_{3}\right)=0.6$, and $l_{i}\left(T|\theta_{3}\right)=0.4$, for i=1,2,3. And for i=4,5,6,7, we assume that $l_{i}\left(H|\theta_{2}\right)=0.7$, $l_{i}\left(T|\theta_{2}\right)=0.3$, $l_{i}\left(H|\theta_{1}\right)=l_{i}\left(H|\theta_{3}\right)=0.2$, and $l_{i}\left(T|\theta_{1}\right)=l_{i}\left(T|\theta_{3}\right)=0.8$. To be clear, we denote the discrete probability distribution that agents 1–3 follow as $L_{1-3}=\left[\begin{array}{ccc} 0.2 & 0.2 & 0.6\\ 0.8 & 0.8 & 0.4 \end{array}\right]$, while agents 4–7 follow discrete probability distribution $L_{4-7}=\left[\begin{array}{ccc} 0.2 & 0.7 & 0.2\\ 0.8 & 0.3 & 0.8 \end{array}\right]$. In this example, successful learning is considered to be reached if $\sum_{i=1}^{7}\left|\mu_{i,t}\left(\theta^{*}\right)-1\right|⩽10^{-3}$. If the collective consensus is not reached after 100 iterations, the learning ends on its own. Results in Figure 1b,c illustrate that an unconnected hypergraph structure may finally result in inconsistent learning results, which means the HSL strategies may not converge in such a case. This phenomenon accords with the intuition that completely separate groups may not reach a consensus.

However, by only adding a hyperedge linking Agent 1 and Agent 4—see Figure 1d—the hypergraph becomes connected. HSL strategies soon converge after a few iterations—see Figure 1e,f—validating the theoretical results in Theorem 1 and the effectiveness of the proposed algorithms.

### 4.2. Hypergraph Structure vs. Convergence Rate

**Figure 2:**
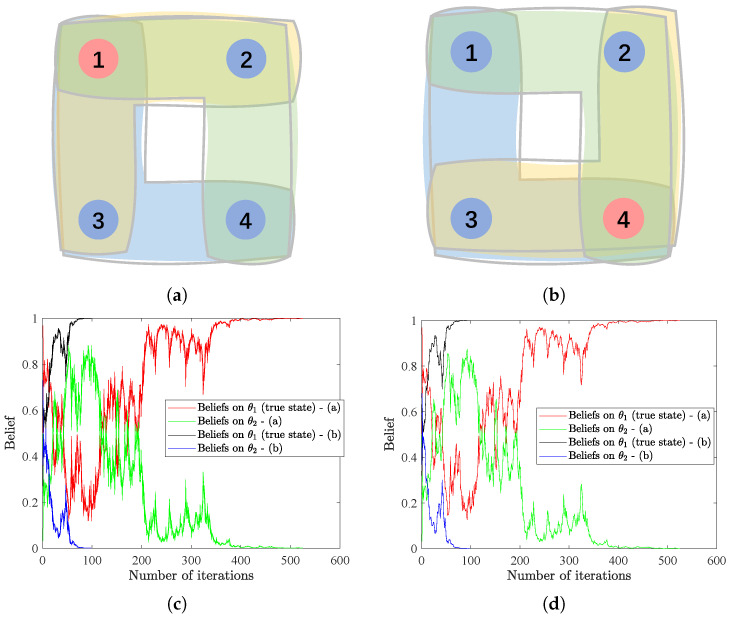
(**a**,**b**) The two hypergraph structures used in this example. (**c**,**d**) The evolution of beliefs on all possible states, with (**c**,**d**) denoting the results of HSL-NEB and HSL-BNE, respectively.

As indicated in Theorem 1 and Corollary 1, the convergence rate is closely related to the eigenvector centrality of matrix *C* as well as the KL-divergence $K(\theta^{*},\theta )$. This offers us the opportunity to increase the convergence rate via assigning a more important role, i.e., a larger eigenvector centrality, to the agent that is more informative, i.e., more helpful to distinguish between true and wrong states. We illustrate this idea by considering two similar hypergraphs shown in Figure 2a,b with four agents and three hyperedges. We define the weight matrices *A* and *B* in the same way as in Remark 1, i.e., for the hypergraph in Figure 2a,

$$A_{l}=\left[\begin{array}{cccc} \frac{1}{3} & \frac{1}{3} & \frac{1}{3} & 0\\ \frac{1}{3} & \frac{1}{3} & 0 & \frac{1}{3}\\ \frac{1}{3} & 0 & \frac{1}{3} & \frac{1}{3} \end{array}\right],\;B_{l}=\left[\begin{array}{ccc} \frac{1}{3} & \frac{1}{3} & \frac{1}{3}\\ \frac{1}{2} & \frac{1}{2} & 0\\ \frac{1}{2} & 0 & \frac{1}{2}\\ 0 & \frac{1}{2} & \frac{1}{2} \end{array}\right],$$

and for the hypergraph in Figure 2b,

$$A_{r}=\left[\begin{array}{cccc} \frac{1}{3} & \frac{1}{3} & 0 & \frac{1}{3}\\ \frac{1}{3} & 0 & \frac{1}{3} & \frac{1}{3}\\ 0 & \frac{1}{3} & \frac{1}{3} & \frac{1}{3} \end{array}\right],\;B_{r}=\left[\begin{array}{ccc} \frac{1}{2} & \frac{1}{2} & 0\\ \frac{1}{2} & 0 & \frac{1}{2}\\ 0 & \frac{1}{2} & \frac{1}{2}\\ \frac{1}{3} & \frac{1}{3} & \frac{1}{3} \end{array}\right].$$

Clearly, Agents 1 and 4 have the largest eigenvector centrality, respectively, for hypergraphs in Figure 2a,b, demonstrating their important roles in the structures. We assume that there are two possible states $\Theta =\{\theta_{1},\theta_{2}\}$, with $\theta_{1}$ being the true state. The initial beliefs are uniformly generated from the interval (0,1) and subject to $\sum_{k=1}^{2}\mu_{i,0}\left(\theta_{k}\right)=1,\forall i=1,\cdots ,4$. At each time step *t*, signal $s_{t}$ is randomly generated following normal distribution N(0,1) and observed by all agents. As $\theta_{1}$ is the underlying true state, from assumptions, we have $l_{i}\left(\cdot |\theta_{1}\right)=N\left(0,1\right),\forall i=1,\cdots ,4$. The likelihood functions of the other state are assigned as $l_{i}\left(\cdot |\theta_{2}\right)=N\left(\frac{i}{10},1\right),\forall i=1,\cdots ,4$, while $\theta^{*}=\theta_{1}$ is globally identifiable. These settings lead to $K_{4}\left(\theta^{*},\theta \right)>K_{3}\left(\theta^{*},\theta \right)>K_{2}\left(\theta^{*},\theta \right)>K_{1}\left(\theta^{*},\theta \right)>0$, depicting that Agent 4 is the most informative agent and Agent 1 is the least informative one. We focus on the number of iterations for update rules HSL-NEB and HSL-BNE to collectively learn the true state, where successful learning is considered to be reached if $\sum_{i=1}^{4}\left|\mu_{i,t}\left(\theta^{*}\right)-1\right|⩽10^{-3}$. Results in Figure 2c,d illustrate that consensus can be significantly faster reached with the structure in Figure 2b, where Agent 4 has both higher eigenvector centrality and is more informative, showing consistency with the theoretical analyses. Moreover, from Lemma 1, the convergence rate of the proposed algorithms and the convergence rate of transition matrix *C* are related to the second largest eigenvalue of *C*. That is to say, the convergence rate depends on the network sparsity (and the signal structures) instead of its size, which means no scaling problem exists for very large networks. However, practically, due to computational burden, we would like to accelerate the convergence rate to reach consensus.

### 4.3. Hypergraph Structure vs. Graph Structure

Here, we provide a preliminary survey on the relationship between traditional social learning on graphs and our HSL framework. As presented in Appendix A.2, every hypergraph H can be mapped to a corresponding graph G by connecting all pairs of nodes that belong to each hyperedge. This method is called *clique expansion* in the field of hypergraph representation learning [52]. We demonstrate the equivalence of a hypergraph and its clique expansion when performing HSL and traditional social learning, respectively, on them theoretically in Appendix A.2 and numerically as described further.

**Figure 3:**
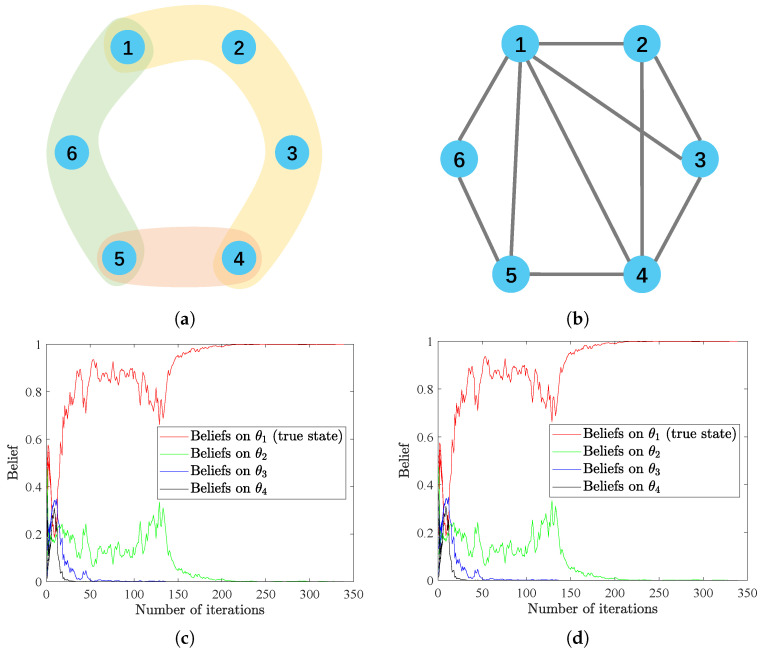
(**a**,**b**) The hypergraph and graph structures used in this example. (**c**,**d**) The evolution of beliefs on all possible states for agents interacting in structures of (**a**,**b**), respectively, with the HSL-NEB algorithm.

We consider a hypergraph with six vertices and three hyperedges; the structure is depicted in Figure 3a. We define the weight matrices *A* and *B* in the same way as in Remark 1, i.e.,

$$A=\left[\begin{array}{cccccc} \frac{1}{4} & \frac{1}{4} & \frac{1}{4} & \frac{1}{4} & 0 & 0\\ 0 & 0 & 0 & \frac{1}{2} & \frac{1}{2} & 0\\ \frac{1}{3} & 0 & 0 & 0 & \frac{1}{3} & \frac{1}{3} \end{array}\right],\;B=\left[\begin{array}{ccc} \frac{1}{2} & 0 & \frac{1}{2}\\ 1 & 0 & 0\\ 1 & 0 & 0\\ \frac{1}{2} & \frac{1}{2} & 0\\ 0 & \frac{1}{2} & \frac{1}{2}\\ 0 & 0 & 1 \end{array}\right].$$

In this way, the matrix C=AB can be written as

$$C=\left[\begin{array}{cccccc} \frac{7}{24} & \frac{1}{8} & \frac{1}{8} & \frac{1}{8} & \frac{1}{6} & \frac{1}{6}\\ \frac{1}{4} & \frac{1}{4} & \frac{1}{4} & \frac{1}{4} & 0 & 0\\ \frac{1}{4} & \frac{1}{4} & \frac{1}{4} & \frac{1}{4} & 0 & 0\\ \frac{1}{8} & \frac{1}{8} & \frac{1}{8} & \frac{3}{8} & \frac{1}{4} & 0\\ \frac{1}{6} & 0 & 0 & \frac{1}{4} & \frac{5}{12} & \frac{1}{6}\\ \frac{1}{3} & 0 & 0 & 0 & \frac{1}{3} & \frac{1}{3} \end{array}\right].$$

The structure of the clique expansion is shown in Figure 3b, and we assume the weight matrix of this graph is identical to *C*. AWe asume there are four possible states $\Theta =\{\theta_{1},\theta_{2},\theta_{3},\theta_{4}\}$ with true state $\theta^{*}=\theta_{1}$. The initial beliefs are uniformly generated from the interval (0,1) and subject to $\sum_{k=1}^{4}\mu_{i,0}\left(\theta_{k}\right)=1,\forall i=1,\cdots ,6$. At each time step *t*, signal $s_{t}$ is randomly generated, following normal distribution N(0,1) and observed by all agents. As $\theta_{1}$ is the underlying true state, from assumptions, we have $l_{i}\left(\cdot |\theta_{1}\right)=N\left(0,1\right),\forall i=1,\cdots ,6$. Moreover, all agents in the network are assumed to be equivalently informative to the true state, with $l_{i}\left(\cdot |\theta_{k}\right)=N\left(\frac{k-1}{5},1\right),\forall i=1,\cdots ,6$ and ∀k=2,3,4. For the hypergraph, we use the proposed update rules HSL-NEB and HSL-BNE, while the LoAB and BLoA algorithms are applied to the graph. In this example, we still regard $\sum_{i=1}^{6}\left|\mu_{i,t}\left(\theta^{*}\right)-1\right|⩽10^{-3}$, the same criterion as in the previous examples as the sign of achieving successful social learning.

Results in Figure 3c,d demonstrate that convergence to collective consensus are reached within almost the same number of iterations for the two cases, i.e., equivalent convergence rate. However, compared to the pairwise communications in graphs, which occur many times in one iteration, HSL significantly reduces the number of mutual interactions among agents and keeps the same convergence rate.

### 4.4. Application to Sensor Cooperative Positioning

**Figure 4:**
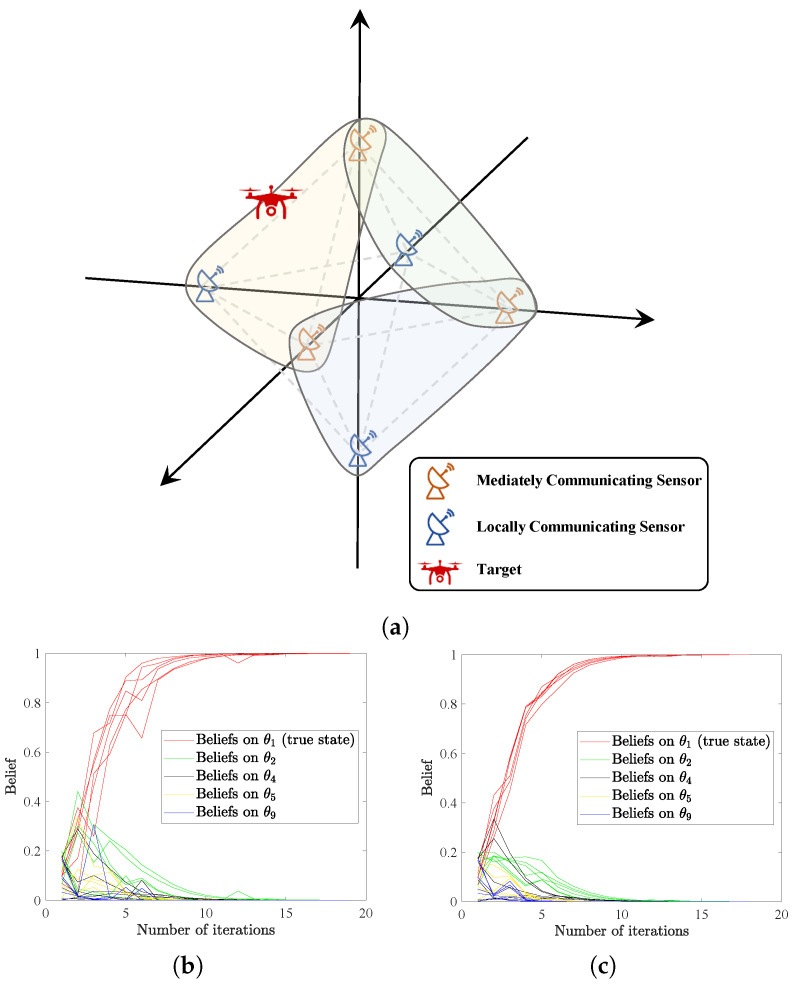
(**a**) The radar network with hypergraph topology considered in this example. (**b**,**c**) The evolution of beliefs on selected states, with (**b**,**c**) denoting the results of HSL-NEB and HSL-BNE, respectively.

In the following example, we demonstrate the applicability of our HSL algorithms in a sensor cooperative positioning problem [29]. We consider a radar network with six sensors located at $\left(\pm 1,0,0\right)$, $\left(0,\pm 1,0\right)$ and $\left(0,0,\pm 1\right)$. The sensors can communicate according to three hyperedge connections, as depicted in Figure 4a, where the *mediately communicating sensor* refers to the sensor belonging to multiple hyperedges, representing its role of communicating across hyperedges, and the *locally communicating sensor* denotes the sensor only communicating in one hyperedge. With this setting, the non-zero elements in the weight matrices *A* and *B* are randomly generated from the interval (0,1) and then normalized to satisfy the row-stochastic assumption. We assume each sensor can sense the target’s location along one dimension only, whereas the target location is a point in a three-dimensional space. Specifically, sensors located on the *x*-axis can sense whether the *x*-coordinate of the target lies in the $\left(-1,0\right)$ or $\left[0,1\right)$ or $\left(-\infty ,-1\right]\cup \left[1,\infty \right)$ interval. Similarly, sensors on the *y*-axis and the *z*-axis can each distinguish between three distinct non-intersecting intervals on the corresponding axis. Therefore, the total number of possible states is nine, i.e., $\Theta =\{\theta_{1},\theta_{2},\cdots ,\theta_{9}\}$, where $\theta_{9}$ denotes the outside infinite region.

The goal is to ascertain the area of the target aircraft by the HSL algorithms. We set $\theta_{1}$ as the true position of the target. The initial beliefs are generated from the interval (0,1) and subject to $\sum_{k=1}^{9}\mu_{i,0}\left(\theta_{k}\right)=1,\forall i=1,\cdots ,6$. Signals are assumed to be generated at each time from set $\left\{\mathrm{NEAR},\mathrm{MID},\mathrm{FAR}\right\}$. Sensors are assumed to be more sensitive to near target, e.g., for sensor 5 on the positive *z*-axis. We set $\left[\begin{array}{c} l_{5}\left(\mathrm{NEAR}|\theta_{k}\right)\\ l_{5}\left(\mathrm{MID}|\theta_{k}\right)\\ l_{5}\left(\mathrm{FAR}|\theta_{k}\right) \end{array}\right]=\left[\begin{array}{c} 0.9\\ 0.05\\ 0.05 \end{array}\right]$ for k=1,2,3,4, and $\left[\begin{array}{c} l_{5}\left(\mathrm{NEAR}|\theta_{k}\right)\\ l_{5}\left(\mathrm{MID}|\theta_{k}\right)\\ l_{5}\left(\mathrm{FAR}|\theta_{k}\right) \end{array}\right]=\left[\begin{array}{c} 0.1\\ 0.85\\ 0.05 \end{array}\right]$ for k=5,6,7,8. Here, *z*-coordinates of $\theta_{1}∼\theta_{4}$ lie in the interval $\left[0,1\right)$, and $\left(-1,0\right)$ for $\theta_{5}∼\theta_{8}$. We additionally set $\left[\begin{array}{c} l_{i}\left(\mathrm{NEAR}|\theta_{9}\right)\\ l_{i}\left(\mathrm{MID}|\theta_{9}\right)\\ l_{i}\left(\mathrm{FAR}|\theta_{9}\right) \end{array}\right]=\left[\begin{array}{c} 0.1\\ 0.1\\ 0.8 \end{array}\right],\forall i=1,2,\cdots ,6$. For other sensors, the signal structures are set up with the same logic. We further assume that the signals are generated following the same probability distribution as the signal structure of $\theta_{1}$. Successful learning is considered to be reached if $\sum_{i=1}^{6}\left|\mu_{i,t}\left(\theta^{*}\right)-1\right|⩽10^{-3}$.

Results in Figure 4b,c illustrate that the true collective consensus can be reached in less than 20 iterations, showing that the sensor network completes the positioning task efficiently and accurately by applying our HSL algorithms. In real scenarios, radar networks always suffer from resource waste due to redundant and repetitive communications especially when using traditional graph-based social learning algorithms. However, in our HSL framework, for example, in the sensor network considered here, the communication can be further reduced to one hyperedge covering sensors on *x*, *y* and *z*-axes, which is enough to guarantee successful positioning.

### 4.5. Application to Consensus in Social Network

**Figure 5:**
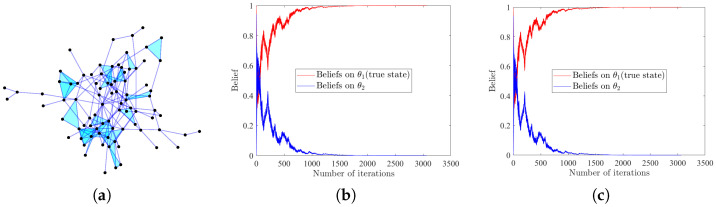
(**a**) Visualization of the real-world hypergraph network Hypertext2009. Shaded triangles denote three-body interactions in the dataset. (**b**,**c**) The evolution of beliefs on all possible states, with (**b**,**c**) denoting the results of HSL-NEB and HSL-BNE, respectively.

We validate the effectiveness of our HSL algorithms in a large-scale real-world higher-order social network, which is often used to model opinion formation [53]. Network topology is determined from public dataset Hypertext2009 provided by the SocioPatterns research collaboration (http://www.sociopatterns.org/). Three-body interactions are reconstructed by Wang et al. [54]. This dataset describes the scenario in which 85 participants exchange their views in a conference in order to form a consensus, e.g., to support or oppose a bill. We assume environmental information can be received by all participants and influences their beliefs. The network structure, consisting of 85 vertices and 225 hyperedges (192 edges + 33 triangles), are given in Figure 5a. The non-zero values in the weight matrices *A* and *B* are randomly generated from $\left(0,1\right)$ and then normalized to satisfy the row-stochastic assumption. We assume there are two possible states $\Theta =\{\theta_{1},\theta_{2}\}$, with $\theta_{1}$ being the true state. The initial beliefs are also uniformly generated from the interval $\left(0,1\right)$ and subject to $\mu_{i,0}\left(\theta_{1}\right)+\mu_{i,0}\left(\theta_{2}\right)=1,\forall i=1,\cdots ,85$. Signals are assumed to be generated randomly, following the Gaussian distribution N(0,1) and observed by all agents. As $\theta_{1}$ is the underlying true state, we have $l_{i}\left(\cdot |\theta_{1}\right)=N\left(0,1\right),\forall i=1,\cdots ,85$. Moreover, the likelihood functions of the other state are assigned as $l_{i}\left(\cdot |\theta_{2}\right)=N\left(\frac{i}{1000},1\right),\forall i=1,\cdots ,85$. We still use $\sum_{i=1}^{85}\left|\mu_{i,t}\left(\theta^{*}\right)-1\right|⩽10^{-3}$ as the sign of successful social learning. Results in Figure 5b,c illustrate that influenced by environmental signals and interpersonal communications, all participants finally reach a consensus and make the right decision, demonstrating the broad applicability of our HSL strategies.

## 5. Discussion and Conclusions

**Figure 6:**
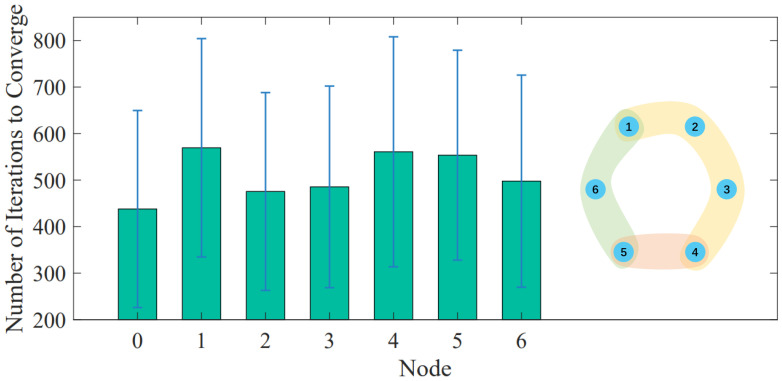
The histogram showing the number of iterations to reaching consensus when the corresponding node is blocked from sensing external information. Here, node “0” represents the plain result with original settings. Error bars denote the standard deviations over 1000 realizations. Notice that blocking the sensors located at mediate position across hyperedges has significantly higher numbers of iterations than blocking sensors lying only in one hyperedge.

Practically, in a sensor network, units may inevitably encounter sudden disconnection due to obstruction, prolonged operations, environmental damages, etc. [55,56]. Here, we discuss a scenario when a sensor loses its ability to sense environmental information. Following the design of the hypergraph considered in Section 4.3, including the network structure, weight matrices, possible states, signal structures, etc., we further assume one of the sensors is blocked from external information and examine the convergence rate, respectively. As shown in Figure 6, where node “0” represents the plain result with original settings and others represent the results with the corresponding node being blocked, the numbers of iterations to reaching consensus all increase when one sensor loses sensing ability. Interestingly, the sensors exhibit different roles in the network; the blocking of Sensors 1, 4 and 5 located at a mediate position across the hyperedges has significantly higher numbers of iterations than the blocking of Sensors 2, 3 and 6 lying only in one hyperedge. This phenomenon reveals that the mediately communicating sensors are more sensitive to environmental obstruction and should be providently cared. In future works, we will comprehensively discuss the relationship between network structure and convergence efficiency.

**Figure 7:**
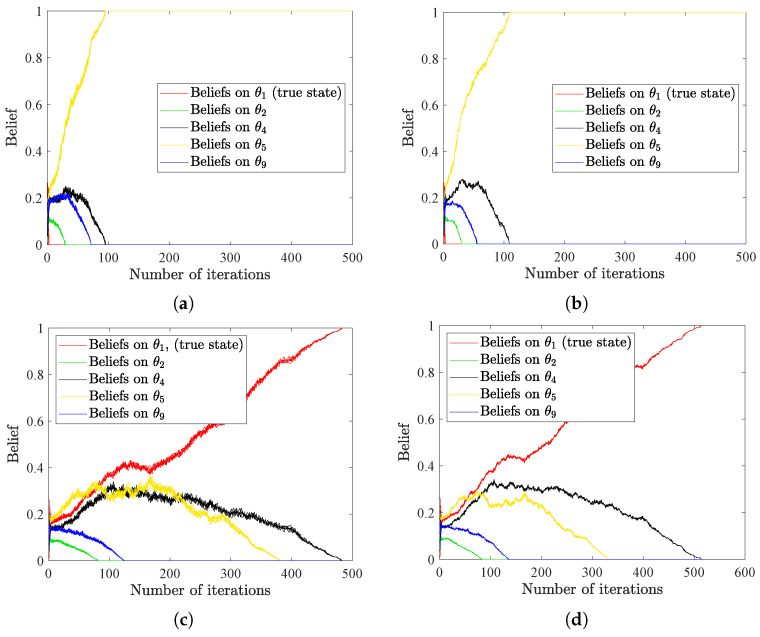
(**a**,**b**) The evolution of beliefs on selected states under bandwidth limit of 6 bits, with (**a**,**b**) denoting the results of HSL-NEB and HSL-BNE, respectively. (**c**,**d**) The evolution of beliefs on selected states under bandwidth limit of 8 bits, with (**c**,**d**) denoting the results of HSL-NEB and HSL-BNE, respectively.

In real-world scenarios, communication constraints seriously influence sensor networks. Based on the radar network considered in Section 4.4, we further introduce bandwidth limitation to the example. We assume that the unit of bandwidth is bits per hypothesis per unit time (we call it bits for short in the following), and the belief on each hypothesis $\theta_{k}$ (k=1,2,⋯,9) is of size *I* bits. Therefore, by adjusting the value of *I*, we can investigate the effect of bandwidth limitation on HSL algorithms. Moreover, to simulate a highly disturbed environment, we choose the signal structures for Sensor 5 on the positive *z*-axis to be $\left[\begin{array}{c} l_{5}\left(\mathrm{NEAR}|\theta_{k}\right)\\ l_{5}\left(\mathrm{MID}|\theta_{k}\right)\\ l_{5}\left(\mathrm{FAR}|\theta_{k}\right) \end{array}\right]=\left[\begin{array}{c} 0.36\\ 0.32\\ 0.32 \end{array}\right]$ for k=1,2,3,4 and $\left[\begin{array}{c} l_{5}\left(\mathrm{NEAR}|\theta_{k}\right)\\ l_{5}\left(\mathrm{MID}|\theta_{k}\right)\\ l_{5}\left(\mathrm{FAR}|\theta_{k}\right) \end{array}\right]=\left[\begin{array}{c} 0.33\\ 0.35\\ 0.32 \end{array}\right]$ for k=5,6,7,8. We additionally set $\left[\begin{array}{c} l_{i}\left(\mathrm{NEAR}|\theta_{9}\right)\\ l_{i}\left(\mathrm{MID}|\theta_{9}\right)\\ l_{i}\left(\mathrm{FAR}|\theta_{9}\right) \end{array}\right]=\left[\begin{array}{c} 0.33\\ 0.33\\ 0.34 \end{array}\right],\forall i=1,2,\cdots ,6$. For other sensors, the signal structures are recomposed with the same logic. Results in Figure 7a,b illustrate that wrong consensus ($\theta_{5}$ in this example) is finally reached when limiting the bandwidth to 6 bits. When raising the bandwidth to 8 bits, as shown in Figure 7c,d, our algorithms can converge to the true collective consensus ($\theta_{1}$ in this example). These results show that HSL algorithms have strong robustness; however, they inevitably make mistakes when the sensor networks suffer from severe interference and limited communication.

There are practically numerous scenarios in which agents demonstrate intentional misbehavior or function in a faulty manner. Extensive studies have been conducted on the robustness of graph-based social learning against Byzantine attacks, wherein adversaries are capable of deviating the system from the prescribed protocol in an arbitrary fashion [57,58]. Nevertheless, the Byzantine attacks on higher-order-based social learning remain unexplored. Our subsequent study will encompass the examination of this particular case.

To conclude, we proposed a new non-Bayesian social learning strategy named the hypergraph social learning by introducing the higher-order topology as the underlying network structure, with its convergence as well as the convergence rate theoretically analyzed. Several numerical examples were provided to demonstrate the effectiveness of the HSL framework and reveal its superior performance when applying it to sensor networks in tasks such as cooperative positioning. Insights regarding the relationship between convergence rate and different positions in the network were given. This work can assist sensor network designers to develop more efficient communication topology, which can better resist environmental obstructions. The HSL framework also has theoretical and applied values in broad areas such as distributed parameter estimation, dispersed information aggregation and social networks. Future works include extending the framework to more complex topologies, such as multi-layer structures, and considering more realistic difficulties in sensor networks such as energy limitations, connectivity loss and inadequate coverage.

## Data Availability

Data sharing is not applicable to this article as no new data were created or analyzed in this study.

## Appendix A. Properties of the Matrix *C*

We prove here that the matrix *C* is the transition matrix of an aperiodic and irreducible Markov chain of finite states under the assumption that hypergraph H is connected without any isolated vertex.

### Appendix A.1. Aperiodicity

From the definition in Assumption 1, $c_{ii}=\sum_{j=1}^{m}b_{ij}a_{ji},\forall i=1,\cdots ,n$. It is clear that if $i\in e_{j}$ (or $e_{j}\in d_{i}$), we have $b_{ij}\neq 0$ and $a_{ji}\neq 0$, and vice versa. Therefore, unless ∃i such that $d_{i}=\emptyset$, we have $\sum_{j=1}^{m}b_{ij}a_{ji}=\sum_{e_{j}\in d_{i}}b_{ij}a_{ji}>0$. The assumption that no isolated vertex exists in H guarantees that $d_{i}\neq \emptyset ,\forall i=1,\cdots ,n$, which leads to $c_{ii}>0,\forall i=1,\cdots ,n$ and the Markov chain is aperiodic.

### Appendix A.2. Irreducibility

For $c_{ij}=\sum_{k=1}^{m}b_{ik}a_{kj}$, where i≠j, we have $c_{ij}=\sum_{e_{k}\in d_{i}\cap d_{j}}b_{ik}a_{kj}>0$ unless ∃i≠j such that $d_{i}\cap d_{j}=\emptyset$. We define graph $G=(V,E^{\prime })$, where V={1,2,⋯,n} and $E^{\prime }=\left\{\left(i,j\right)|d_{i}\cap d_{j}\neq \emptyset \right\}$ and $M={\left(m_{ij}\right)}_{n\times n}$ is the transition matrix corresponding to G. Clearly, $m_{ij}>0$ is equivalent to $d_{i}\cap d_{j}\neq \emptyset$. Therefore, the connectivity of H induces the connectivity of G, which further ensures that *C* is irreducible.
